# Recovery of an Antioxidant Derived from a Phenolic Diphosphite from Wastewater during the Production of a Polypropylene Compound: A Step towards Sustainable Management

**DOI:** 10.3390/molecules29122780

**Published:** 2024-06-11

**Authors:** Joaquín Hernández-Fernández, Elias Bello-Leon, Juan Carrascal

**Affiliations:** 1 Chemistry Program, Department of Natural and Exact Sciences, San Pablo Campus, University of Cartagena, Cartagena 30015, Colombia; 2 Chemical Engineering Program, School of Engineering, Industrial and Technological Park Carlos Vélez Pombo, Technological University of Bolivar, Km 1 Vía Turbaco, Turbaco 130001, Colombia; 3 Department of Natural and Exact Science, Universidad de la Costa, Barranquilla 30300, Colombia; 4 Science and Engineering Research Group CECOPAT&A, Chemistry Program, Department of Natural and Exact Sciences, San Pablo Campus, University of Cartagena, Cartagena 131001, Colombia; ebellol@unicartagena.edu.co; 5 Research Group GIA, Comfenalco Technological University Foundation, Cartagena 30015, Colombia; invest.ambiental@tecnologicocomfenalco.edu.co

**Keywords:** extraction, organic, antioxidants phenolics phosphoesters, polypropylene, industrial, wastewater

## Abstract

Organic phosphoester (OPE) antioxidants are currently required due to their contribution to enhancing the quality of polymers, including polypropylene (PP). In this research, an integral methodology is presented for the efficient extraction of bis(2,4-dicumylphenyl) pentaerythritol diphosphite from industrial wastewater. Upon employing the solid-phase extraction (SPE) technique, the recovered compound is subjected to a comprehensive analysis of the recovered compound using high-performance liquid chromatography (HPLC), mass spectrometry (MS), thermal analysis (TGA), Fourier transforms infrared spectroscopy (FTIR), and differential scanning calorimetry (DSC). Subsequently, purified Bis(2,4-dicumylphenyl) pentaerythritol diphosphite was evaluated as a thermo-oxidative stabilizer after incorporation into PP resins. The relative standard deviation (RSD), Error (Er), linearity (R^2^), and percentage (%) recovery were less than 2.6, 2.5, more significant than 0.9995, and greater than 96%, respectively, for the inter-day and intra-day tests of the chromatographic method and the SPE. Except for chloroform, which was necessary due to the solubility properties of the investigated analyte, the use of environmentally friendly solvents, such as methanol and acetonitrile, was considered during the development of this research. The OPE extracted from industrial wastewater was characterized by FTIR, UV–Vis, DSC, TGA, and MS, allowing the elucidation of the structure of Bis(2,4-dicumylphenyl) pentaerythritol diphosphite (BDPD). The recovered OPE was mixed with PP resins, allowing it to improve its thermal properties and minimize its thermo-oxidative degradation. Organophosphorus flame retardant (OPE)’ concentration in wastewater is alarming, ranging from 1179.0 to 4709.6 mg L^−1^. These exceed toxicity thresholds for aquatic organisms, emphasizing global environmental risks. Using a validated solid-phase extraction (SPE) technique with over 94% recovery, the study addresses concerns by removing organic contaminants and supporting circular economy principles. The high economic and environmental significance of recovering BDPD underscores the need for urgent global attention and intervention.

## 1. Introduction

The large volumes of waste produced by companies these days, particularly chemical compounds with fast production rates, are a major source of worry and call for an assessment of their environmental effects [1,2,3,4]. The industries producing plastic materials fit into these significant contributors of waste of interest, since for the year 2020, only in Europe, approximately 2.51 million tons (MT) were generated, of which only 7.6% were recovered [5]. However, a significant volume of 260 thousand tons (kT) of plastic was identified in the oceans [6,7,8]. One of the most produced polymers is polypropylene (PP) [9,10,11,12,13,14]. The great concern of plastic is that its entry into the aquatic environment is easy, which is why it is considered an emerging organic pollutant (EOP) [8,15,16,17]. Most of these EOPs are not regulated; however, should the possibility of the harm they might pose to people and other animal and plant species be assessed, they might be candidates for regulation [18,19,20].

The above scenario has prompted several research initiatives focused on the synthesis, characterization, uses, and propagation of EOPs in water bodies during the last two decades [18,19,20]. It is known that many of the additives used in the plastic manufacturing process tend not to be chemically bound to the polymeric matrix that contains them. Therefore, when the polymer comes into contact with water, the EOP can migrate toward other media [21,22,23,24,25]. For some EOPs, the generation of adverse effects in living organisms has been shown, including them in the category of OPE [26,27,28,29]. The migration of these OPEs is accelerated under the influence of different variables such as temperature, hydrogen potential, conductivity, plastic particle size, salinity, and mechanical processes of these plastic residues [30,31,32,33]. The OPEs—specifically, the aryl organic phosphoesters (AOPEs) such as triphenyl phosphate, diphenyl phosphate, and tricresyl phosphates, among others—have toxic effects that cause dysfunction of the endocrine system evident in changes at the hormonal level; dysfunction of the metabolic system of the human body by modifying molecular mechanisms; damage and malfunction of the cardiovascular system; liver tissue damage and malfunction; and headache, anxiety, confusion, and altered state of consciousness among others [34,35]. OPEs can cause considerably more serious damage in children than in adults, and OPEs have even been found in the placenta of pregnant women. Two of the most notorious adverse effects in children exposed to these substances are poor neurological development and asthma [36]. One of the additives commonly used for this purpose in plastics is BDPD (Bis(2,4-dicumylphenyl) pentaerythritol diphosphite) [36,37]. This is an OPE additive that is used as a thermal stabilizer, antioxidant, and flame retardant [38], given the presence of a phosphorus atom, phosphite form, and aromatic rings with steric hindrances that prevent multiple reactions of the phenol group in its chemical structure [39]. This OPE needs to present relevant studies, given its recent application in polymeric matrices, its large volumes of consumption, and its structural correlation with other synthetic antioxidants that allow it to be classified as a molecule with significant potential to generate adverse effects on the environment and global health [26,27,28,29,38,39,40,41,42,43,44]. This is supported by its negative impact on some species, such as zebrafish, daphnia magna, and selanastrum subspicatus, where low concentrations have lethal toxicity. In this sense, this OPE Doverphos presents LC50 [96 h] > 0.22 mg/L, NOEC > 0.22 mg/L for Brachydanio rerio (Zebra fish); EC50 [48 h] > 0.20 mg/L and NOEC = 0.06 mg/L for Daphnia magna; and EbC50 [72 h] > 0.49 mg/L, EµC50 [72 h] > 0.49 mg/L, and NOEC > 0.49 mg/L for Selanastrum subspicatus. OPEs, in general terms, have been studied in agricultural basins, soil, aquatic environments, and homes and supermarkets, which makes their surveillance and control of global interest [35,45,46,47].

Chemical techniques used to extract, identify, detect, and quantify OPEs at different sites include liquid phase microextraction (LPME), solid-phase extraction (SPE), and solid-phase microextraction (SPME) techniques, among others [35,45,46,47], which, combined with instruments such as thermogravimetric analysis (TGA), differential thermal scanning (DSC), Fourier transformed infrared (FTIR), high-performance liquid chromatography (HPLC), and gas chromatography (GC), have allowed obtaining reliable results for the scientific community [38,39,48,49,50,51,52,53,54,55,56].

Solid-phase extraction (SPE) is a vastly significant procedure in the scientific domain owing to its notable selectivity by utilizing cartridges with specific solid phases. This selectivity enables precise extraction of the analytes of interest within a sample. It is crucial to highlight that SPE entails a series of significant advantages compared to other extraction methods. Among these advantages are the reduction in costs associated with eluents and reagents, as well as decreased apparatus usage, while simultaneously achieving higher recovery rates of the desired analytes. Due to its efficiency, the SPE methodology shortens procedural times, ensures excellent safety, and enhances analytical precision in determining the components under study.

Moreover, its broad compatibility with various samples and matrices is notable, allowing its applicability across diverse scientific fields. However, it is essential to underscore some inherent disadvantages of this method. These include the requirement for substantial quantities of solvents, which may present economic and environmental limitations. Additionally, solid-phase extraction may involve extensive time for the complete extraction of analytes, which could be a factor to consider in applications requiring faster turnaround for results [57,58]. 

The research and development of functional chemical compounds are pivotal in continuously improving various industrial applications [59,60]. Within this context, Bis(2,4-dicumilphenyl) pentaerythritol diphosphite (BDPD) emerges as a critical component in stabilizing polymers, imparting essential properties for their performance and durability. However, this compound’s efficient and precise extraction has posed a significant challenge due to its molecular structure [61]. The pressing need to develop trustworthy analytical methods for gathering and measuring BDPD has driven the exploration of advanced techniques in analytical chemistry [61]. In this regard, the current study aims to assess the various state-of-the-art analytical techniques viabilities, including solid-phase extraction (SPE), high-performance liquid chromatography (HPLC), thermogravimetric analysis (TGA), Fourier transform infrared spectroscopy (FTIR), and ultraviolet–visible spectroscopy (UV–Vis), for the effective extraction of BDPD. The primary interest of this study lies in overcoming challenges associated with the selective extraction, precise identification, and reliable quantification of BDPD in complex matrices. The selection of these techniques is based on their potential to offer a comprehensive set of procedures for sample preparation, separation, detection, and molecular characterization, allowing for exhaustive analysis of BDPD in real-world samples with industrial relevance. The success of this research will not only contribute to the development of advanced analytical methodologies but also support the quality and safety of polymer products stabilized with BDPD, facilitating their application in various industries. Furthermore, the knowledge gained from this exploration could have significant implications for optimizing manufacturing processes and formulating high-performance products in the industrial domain.

## 2. Results and Discussion

### 2.1. Improved HPLC Separation

Chloroform, dichloromethane (DCM), hexane, and acetonitrile (ACN) are medium- and low-polarity solvents compatible with BDPD. With C8 and C18 reverse phase columns and high-purity silica columns, these OPEs are usually separated. A Lichrosorb RP-18 column (4.6 m × 200 mm × 5 m) was chosen to separate the OPE of interest in this experiment.

Using the constant flow rate of 1 mL min^−1^, several ACN:H_2_O mobile phase gradients were used, starting with a ratio of 65:35 and ending with 100% ACN. The results revealed inadequate resolution, inefficiently high solvent use, and considerable data collection time. Testing flow rates between 1.2 and 2.0 mL min^−1^ improves peak width and retention times. This investigation reached ratios of 85% ACN and 15% H_2_O (1 min, 1.5 mL min^−1^), 91% ACN and 9% H_2_O (2 min, 2 mL min^−1^), 95% ACN and 5% H_2_O (5 min, 3.5 mL min^−1^), and 100% ACN and 0% H_2_O, allowing suitable chromatographic profiles. The chemical nature of BDPD and the active mobile phase allowed for establishing separation times with adequate chromatographic resolution and eluotropic power.

### 2.2. Optimization of Sample Preparation Procedure

**Figure 1:**
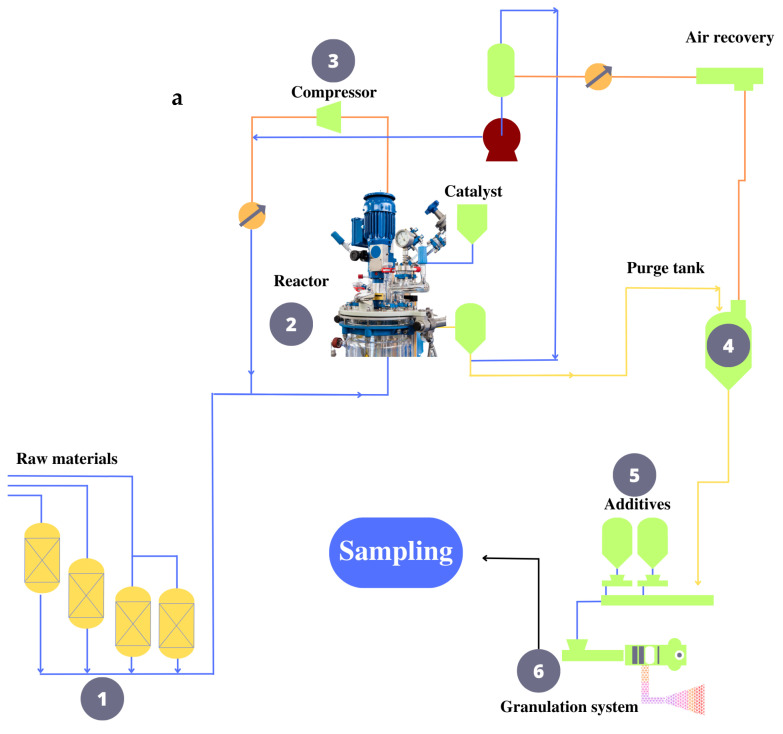

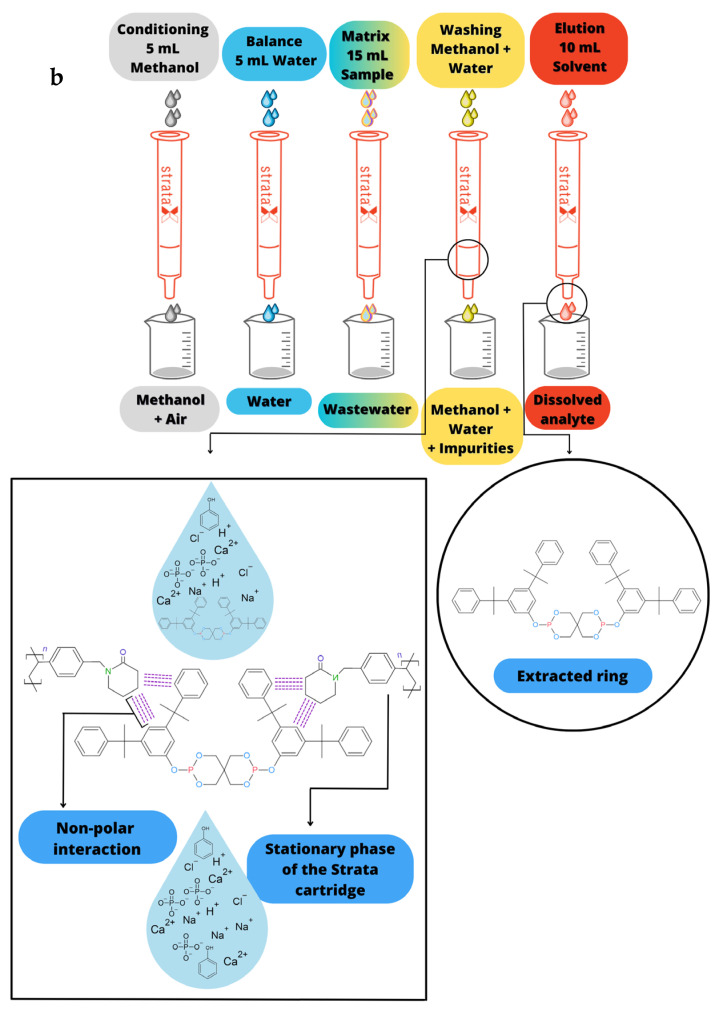
PP production process where the sampling of industrial wastewater is carried out (**a**), and Bis(2,4-dicumylphenyl) pentaerythritol diphosphite extraction process by SPE (**b**).

The wastewater that is sampled in stage 6 of the PP production process, shown in Figure 1b, is characterized by having a petrochemical matrix that has a complicated composition, colorful appearance, two phases, the presence of suspended particles of various sizes, and an unpleasant odor, among other characteristics. These organoleptic characteristics of the sample reveal a very intriguing analytical question since they suggest that the sample matrix contains several characteristics that need to be investigated to avoid possible interferences in the separation. Table 1 records the range intervals for the physicochemical parameters of the industrial wastewater samples studied. All values fall within the limits established by the relevant Colombian technical standard, Resolution 0631 of 2015, which regulates Article 28 of Decree 3930 of 2010 and updates Decree 1594 of 1984.

Thanks to this optimization analysis of the preparation technique of these industrial wastewater samples, it was possible to decide whether to eliminate or isolate the supplied analytes to prevent losing precision or accuracy during this preconcentration stage. This optimization examines SPE conditioning, break volume, elution conditions, wash conditions, and the preconcentration system. The initial step to condition the SPE stationary phase was to add 5 mL of MeOH and then 5 mL of HPLC-grade water. The methodology supplied by Phenomenex (Torrance, CA, USA), manufacturer of the Strata-X 33u, was used as a guide to carry out this conditioning. To ensure the highest dissolution capacity for the analyte of interest, work was performed on selecting the optimal solvent with the proper polarity and the minor amounts. The strategy aims to recover as much Doverphos S-9228 as possible. This approach’s precision, LOD, and LOQ are at risk if this goal is not achieved, as low recovery will lead to a decrease in the analytical signal. Experiments were performed with 100% ACN, searching for the best solvents. As part of the process of determining the best quantities, ml to mL monitoring was used. Ten replicates were performed after discovering that the appropriate amount to elute is 8 mL of ACN. Preconcentration by nitrogen injection to dryness was chosen since our OPE molecule is a diphosphite of molecules with several aromatic rings and steric hindrances. 

To avoid possible thermal deterioration or decomposition of the target analytes, solvent evaporation by heating the plate was ruled out. Instead, evaporation with a stream of N_2_ was used.

### 2.3. Validation of Analytical Methodology

#### 2.3.1. Repeatability and Reproducibility of the Calibration Curve

To identify the repeatability (RPED) of the calibration curve, five tests using the HPLC-MS method, the same standard, the very same day and the same operator were conducted based on the relative standard deviation (RSD) and six concentrations between 0 (blank) and 5000 ppm. If the daily mean values were less than 20%, the precision was deemed verified. An acceptable condition is recommended to be a variation from the anticipated value of fewer than 15% [41,62,63,64]. The precision values and the parameters derived from the acquired values are shown in Table 2. Five tests were conducted with different operators on separate and consecutive dates, using the same analytical technique and standard, to ascertain the repeatability (RPOD) of the calibration curve. 

#### 2.3.2. Repeatability and Reproducibility of SPE

The precision of the SPE system for the cleaning and extraction of the BDPD was evaluated through the study of the RPED. To execute the RPED research, an analysis of six replicates was carried out with concentrations between 0 and 5000 mg L^−1^ on the same day with three different SPE cartridges. The follow-up of the area and retention time of the BDPD resulting from the treatment by the SPE showed variations of less than 3% in area and 2.3% in retention time for the runs carried out on the same date. The set of registered values is established in Table 3. The same operator obtained an RSD of less than 2.3% (1500 and 2000 ppm), error rates lower than 4.4%, and recoveries equivalent to or more significant than 96% of all instances. 

The maximum yield achieved under these circumstances was 98%. We reached a maximum RSD value of 2.1% (1000 ppm) for the RPOD under the mentioned conditions, errors less than 3.4%, and recoveries of at least 96%. The data obtained for the RPED and RPOD of the SPE are established in Table 3.

#### 2.3.3. LOD, LOQ, Linearity, and Range

After injecting and evaluating five samples of the same standard from each of the six points defined in the 1000 to 5000 mg/L range, the straightness of the BDPD calibration curve was ascertained. Table 3 depicts the regression (R^2^) and correlation (r) coefficients, which confirm the linearity and good behavior of the data in the range selected for the chromatographic method. You can understand that an excellent linear behavior occurred; the R^2^ was registered at 0.99979, 0.99979, 0.99989, and 0.99996 for the RPED of the curve, RPOD of the curve, RPED of SPE, and RPOD of SPE, respectively (see Table 4). The LD was 1.3 mg/L and the LOQ was 7.7 mg/L for the BDPD.

The chromatographic method’s threshold of quantifications (LOQs) falls between 5.4 and 16 mg L^−1^ and is lower than the ecotoxicological values disclosed for each chemical compound. Consequently, its use is highly valuable for managing industrial processes and controlling emissions into the environment.

The study focuses on quantifying Doverphos in industrial wastewater, revealing significant concentrations ranging from 1179.0 to 4709.6 mg L^−1^. This substantial presence raises environmental concerns, emphasizing the importance of addressing its elimination or reduction in industrial discharges. However, the presence of Doverphos in such high concentrations raises concerns about its environmental impact, especially on water quality and aquatic ecosystems. This underscores the need to effectively address this contamination and consider measures to mitigate its release in industrial wastewater.

### 2.4. Characterization and Comparison of Pure and Recovered Bis(2,4-Dicumylphenyl) Pentaerythritol Diphosphite

The chemical characterization of BDPD was given using TG, UV–Vis, DSC, FTIR, and MS techniques. The results generally show that the structural and physicochemical properties of BDPD extracted from wastewater are comparable to those of the pure Doverphos molecule. This demonstrates that the extraction process’s method eliminated all extraneous components from the BDPD, resulting in a highly pure BDPD, as shown by the outcomes.

#### 2.4.1. Thermal Stability Characterization of Polymer with Pure and Recovered Bis(2,4-Dicumylphenyl) Pentaerythritol Diphosphite

**Figure 2:**
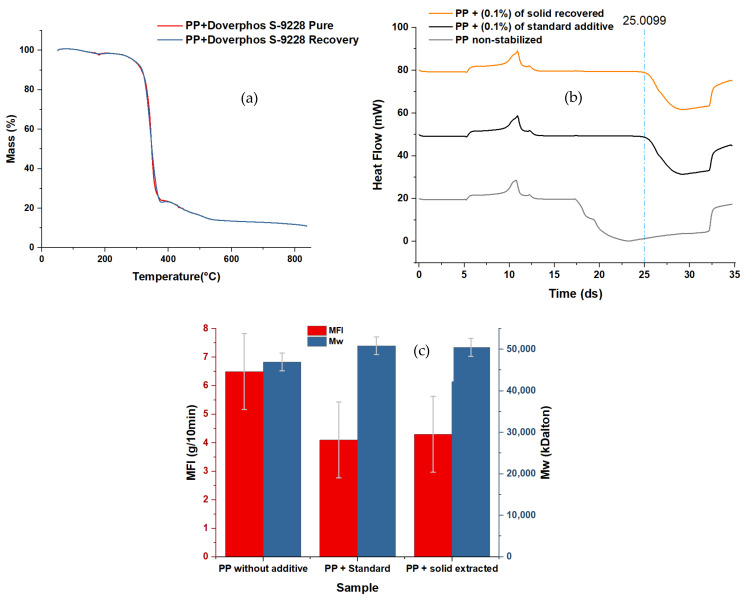
Thermal and mechanical analysis of pure and recovered Doverphos S-9228 by TGA (**a**) and added to a polymeric blend of virgin PP resin (**b**,**c**).

The thermal stability of these compounds was evaluated directly by thermogravimetric analysis (TGA), as represented in Figure 2a. Research on Doverphos is limited. Few studies on BDPD are available [39,64,65,66,67]. Among them, two studies have specifically explored the thermo-oxidative stability provided by BDPD when integrated into polystyrene (PS), employing TG as the analytical method [68,69]. Pure BDPD in the PP matrix showed stability up to 98 °C, followed by a gradual decrease in mass until reaching 269 °C, where a remarkable mass loss started. Between 269 and 380 °C, a substantial mass loss of 72.95% was observed. After that, the sample showed a decreasing mass loss rate; from 380 to 837 °C, an additional mass loss of 11.92% was recorded. The complete thermal analysis covered 51 to 837 °C. 

This detailed analysis reveals the thermal degradation profile of BDPD within the PP matrix, delineating distinct temperature ranges associated with notable diminution in mass. These studies advance our comprehension of this additive’s stability and thermal behavior in PP matrices. 

The thermal degradation characteristics of the retrieved BDPD closely resembled those of pure BDPD. Subtle and negligible alterations were solely evident around 182 °C and between 369 and 392 °C, signifying the remarkable purity of the recovered Doverphos (see Figure 2a). This observation serves as a significant indicator of its favorable performance as an additive for potential reintroduction into polymer matrices, specifically in Polystyrene (PS) and Polypropylene (PP) contexts [68,69].

This congruence in thermal behavior between the recovered BDPD and its pure form underscores its resilience and stability even after retrieval, implying its promising suitability for incorporation as an effective additive in diverse polymer matrices. Such findings substantiate the viability of reintegrating this recovered additive into industrial processes, showcasing its potential for sustainable and efficient utilization in polymer applications. The successful recovery of Doverphos from industrial wastewater presents compelling opportunities within industrial applications. The reintroduction of this recovered compound into manufacturing processes offers a potential avenue for cost savings by reducing the need to acquire new materials. 

Furthermore, its incorporation into polymer and plastic production, particularly in the enhancement of thermal properties in materials like PP resins, signifies a promising approach toward creating more durable and resilient end products. 

This study aligns with broader sustainability initiatives in the chemical industry by advocating for the reuse of Doverphos. By circumventing waste generation and optimizing raw material usage, the reintegration of recovered Doverphos showcases a step towards a more sustainable industrial framework. Moreover, integrating recovered Doverphos into circular economy models embodies a closed-loop system, enabling the compound to be reused across various applications rather than being discarded as waste. This not only mitigates environmental concerns associated with water contamination but also promotes resource efficiency and a more responsible approach to industrial processes.

#### 2.4.2. Analysis of Pure BDPD and Recovered BDPD by UV–Vis Spectrophotometry

**Figure 3:**
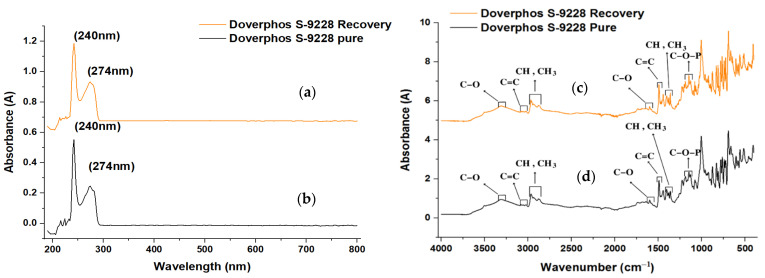
UV–Vis recovered Doverphos (**a**), UV–Vis pure Doverphos (**b**), FTIR recovered Doverphos (**c**), and FTIR pure Doverphos (**d**).

The solubilization of BDPD in chloroform posed challenges due to its inherently low solubility, even within organic solvents. To facilitate solubility, the process involved elevating the temperature to 35 °C for 15 min, aided by ultrasound at a frequency of 60 Hz. Despite efforts, the initial concentrations of 5000, 2000, and 1000 mg L^−1^ of BDPD for UV analysis led to poor resolution, evident by the saturation observed in the absorption spectrum bands (see Figure 3a,b). In response to this, from 1000 mg L^−1^, the initial solution was diluted by a factor of 1/5 to create a fresh solution with a concentration of 200 mg L^−1^. Although this improved the UV–Vis spectrum resolution, it remained suboptimal. Consequently, a further dilution was performed with the same factor, resulting in a new solution of 40 mg L^−1^. At this concentration, distinct bands of maximum absorbance at 240 and 270 nm were observed (Figure 3a,b). The UV–Vis analysis revealed that the absorption profiles of both pure and recovered BDPD were remarkably similar, exhibiting negligible differences that do not significantly impact critical aspects of the analysis. This alignment underscores the efficacy of the removal and recovery process for this antioxidant molecule from wastewater, highlighting an excellent recovery alternative and an efficient environmental impact remediation process.

#### 2.4.3. Infrared Spectrum Analysis for Pure and Recovered Additive

A comprehensive Fourier Transform Infrared Spectroscopy (FTIR) investigation compared the spectra of pure BDPD with the solid recovered from wastewater (refer to Figure 3c,d). The recovered analyte (Figure 3c) and the pure molecule’s spectrum showed remarkable similarities in the FTIR data, suggesting an elevated degree of purity in the recovered Doverphos S-9228. Distinct absorption bands within the spectrum provided valuable insights into the molecular composition. The region spanning 3478 to 3090 cm^−1^ displayed an absorption band associated with ester groups. A notable overlapping absorption band attributed to CH vibrations appeared at 2869 cm^−1^, accompanied by a distinct band corresponding to CH_3_ vibrations at 2960 cm^−1^. At 1398 cm^−1^, a distorted absorption band representative of CH and CH_3_ vibrations was observed.

Moreover, the spectrum revealed characteristic features related to the phosphite group (C-O-P) between 1022 cm^−1^ and 1212 cm^−1^ [30]. Additionally, the C=C aromatic bond presence was discernible, albeit faintly, observed between 3092 and 3000 cm^−1^, 1601 cm^−1^, and 1490 cm^−1^. These distinct absorption bands and their intensities align closely between the pure and recovered Doverphos S-9228, substantiating the efficient recovery and preservation of its chemical composition from the wastewater matrix. This spectral congruence further supports the high purity and integrity of the recovered Doverphos S-9228, establishing its potential utility in various applications despite the extraction process.

#### 2.4.4. Mass Spectrometry for Pure and Recovered Bis(2,4-Dicumylphenyl) Pentaerythritol Diphosphite

**Figure 4:**
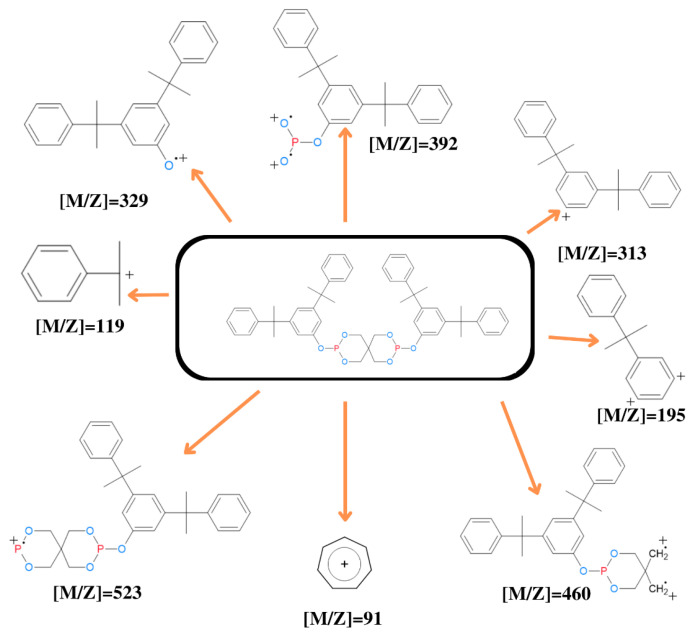
Bis(2,4-dicumylphenyl) pentaerythritol diphosphite fragmentation patterns.

Figure 4 depicts the molecular ion masses derived from the fragmentation of bis(2,4-dicumylphenyl) pentaerythritol diphosphite via mass spectrometry detection. Among these, the most prevalent molecular ions exhibit a ratio *m*/*z* = 91, 119, 195, and 313, indicative of secondary and tertiary carbocations. Notably, these ions are abundant due to their stability in the fragmentation process. The energy required to disrupt the C-C bond is comparatively lower than that needed for breaking the C-O and O-P bonds. The formation of the tropylium ion (*m*/*z* = 91) results from the breakage of the C-C bond inside the tertiary carbon, which splits the aromatic ring’s terminal carbon and the carbon that connects to the oxygen atom and nearby methyl groups. This phenomenon aligns with common ion formations observed in organic molecule fragmentation via mass spectrometry. Conversely, molecular ions with ratios [*m*/*z*] = 329, 392, and 523 exhibit lower abundances due to the inherent instability of the positively charged radical formed within the O+ and P+ clusters. The energy required for bond cleavage involving these atoms (O and P) is not substantially different from that needed for C-C bond cleavage, influencing the lower abundance of these ions. 

The least abundant molecular ion with a ratio [*m*/*z*] = 460 harbors a primary carbocation, which exhibits high instability, thus contributing to its minimal abundance within the spectrum. This observation underscores the influence of molecular structure on the formation and abundance of ions in the mass spectrometry fragmentation process for organic compounds.

#### 2.4.5. Industrial Application of Recovered Bis(2,4-Dicumylphenyl) Pentaerythritol Diphosphite

PP has various applications, such as the manufacture of chairs, plastic caps, bottles, bricks, shoes, and computer protectors. At elevated temperatures ranging from 190 to 250 °C, PP undergoes oxidation, degradation, chain scission, increased fluidity, and substantial alterations in its molecular weight distribution. To assess Bis(2,4-dicumylphenyl) pentaerythritol diphosphate’s effectiveness in the PP matrix, a new end method was explored. To evaluate its impact on PP, oxidative induction time analysis, PP melt index measurements, and an estimation of the molecular weight distribution were performed. The blends were then formed into sheets by compression molding on a CARVER 3895 hot press. This comprehensive analysis aims to evaluate how the inclusion of recovered Bis(2,4-dicumylphenyl) pentaerythritol diphosphite affects PP’s thermal stability, melt behavior, and molecular characteristics. Such assessments are crucial in exploring the potential revitalization of this additive in offering enhanced performance and sustainability in various PP applications subjected to elevated temperatures.

#### 2.4.6. Oxidation Induction Time for Recovered Solid Compared to BDPD Standard

The determination of oxidation time involved analyzing the curves obtained from DSC, correlating expected time against heat flux. Over time, the slope of the curves for PP samples altered, indicating a new exothermic behavior consistent with oxidation, as depicted in Figure 2b. Initially, an endothermic peak was observed, suggesting a preliminary stage before oxidation initiation. Significantly, the oxidation time for unstabilized PP (Figure 2b) was notably shorter compared to PP formulations containing the additive and the recovered BDPD. This oxidation time signifies the moment when the slope shifts, marking the initiation of oxidation within the samples. For the PP blends with the pure additive and the recovered BDPD, the oxidation induction time (OIT) was measured at 8.4 min, underscoring how the presence of the additive and recovered BDPD mitigates PP oxidation and enhances its thermal stability. As can be observed from the change in slope at 25.0 min, there is a substantial distinction between the stabilized and unstabilized PP samples. In sharp contrast, the OIT for the unstabilized PP was a mere 0.65 min. This analysis underscores the pivotal role of the additive and the recovered BDPD in prolonging the onset of oxidation in PP, highlighting their efficacy in bolstering PP’s resistance to thermal degradation. The OIT values serve as crucial indicators of enhanced thermal stability in PP formulations augmented by these additives. 

### 2.5. MFI

Utilizing the melt flow index (MFI) depicted in Figure 2c allows for the assessment of PP fluidity, where higher MFI values correspond to increased molten PP fluidity, indicating a direct proportionality between MFI and fluidity. The findings from the MFI analysis underscore the thermal stabilization effect of the recovered BDPD on PP. Introducing a 0.1% concentration of recovered Doverphos notably enhances the MFI. Unmodified virgin PP exhibits an MFI of 6.6, indicative of its baseline fluidity. The retrieved Doverphos and the pure Doverphos entering the PP matrix demonstrate a decrease in MFI to 4.21 and 4.41 g/10 min, respectively. Notably, the transition from an MFI of 6.6 to 4.21 and 4.41 g/10 min corresponds to molecular weight (Mw) values of 47,148; 51,324; and 51,743 daltons, respectively. The shift in Mw indicates that the molecular weight distribution of the PP blends has undergone a substantial change. The observations reveal that the incorporation of recovered and pure Doverphos into PP leads to a narrower distribution of molecular weights, resulting in a notable increase in Mw. This shift in Mw indicates alterations in the polymer’s molecular structure, potentially contributing to enhanced thermal stability and altered flow characteristics of the PP blends. 

All the results derived from the evaluation of both additives are favorable, with particular emphasis on the recovered additive. When incorporated into the polymeric matrices, this additive fulfills the function of mitigating the oxidative degradation of these matrices, acting as a highly effective antioxidant. In addition, a modification in the ignition temperature for polymer combustion is observed. In addition to its effectiveness as an antioxidant, this additive exhibits the ability to modify the temperature at which the flash point for polymer combustion is reached.

### 2.6. Environmental Impact Estimation

The obtained concentration values varied from 1179.0 to 4709.6 mg L^−1^, 1179.0, and 4709.6 mg L^−1^. These samples had recoveries equal to or greater than 94.31% and RSD less than 5%. These OPE concentration levels in wastewater are very high. This industrial water is considered to have very high levels of environmental impact on human health since the measured concentrations are higher than those of other aryl organic phosphoesters (AOPEs) such as triphenyl phosphate, diphenyl phosphate, and tricresyl phosphates, among others, which have toxic effects causing dysfunction of the endocrine system; dysfunction of the human body’s metabolic system; damage and malfunction of the cardiovascular system and the liver tissue; and headache, anxiety, confusion, and altered state of consciousness, among others [34].

These high concentrations are alarming and generate national and international alarm as these OPEs are used in petrochemical plants that produce polymers worldwide. It should be noted that in South America, North America, Europe, Asia, etc., petrochemical plants use this OPE to increase the thermal stability of their products. In the chemical process of this investigation, the sampling point and the point where the residual water is generated are observed. Therefore, petrochemical plants in the rest of the world are expected to generate this wastewater, which can generate similar environmental impacts. It is noted that the concentrations of this OPE in this investigation are higher than the LC50 (96 h, >0.22 mg/L) and NOEC (0.22 mg/L) for Brachydanio rerio (zebrafish). The concentrations of this OPE that we have found in these wastewaters from this investigation are also higher than the EC50 (48 h, >0.20 mg/L) and the NOEC (0.06 mg/L) for Daphnia magna. Furthermore, it can be seen that the impact ranges of this OPE in this investigation are much higher than EbC50 (72 h, >0.49 mg/L), EµC50 (72 h, >0.49 mg/L), and NOEC (>0.49 mg/L) for Selanastrum subspicatus [36,46,47,48]. It is crucial to mention that this compound has been considered by the Globally Harmonized System (GHS) under code P273, which stipulates the imperative to prevent the release of this substance into the environment [70,71]. Given the limited or non-existent information reported about the BDPD production process, conducting a life cycle assessment (LCA) was impossible. For the remeasurement of these high levels of OPE, the previously validated SPE technique is applied, and it was possible to demonstrate recovery efficiencies greater than 94%. This makes it possible to eliminate the organic load of this industrial wastewater and recover the OPE to give an industrial application that supports the sustainability and circular economy of these industrial processes. Table 5 summarizes the potential damages that BDPD in wastewater could cause when discharged into natural bodies of water.

## 3. Materials and Methods

### 3.1. Reagents

Merck provided the acetonitrile (HPLC grade) and methanol (MeOH) for the experiment (Darmstadt, Germany). Ammonium hydrochloride at 99.5% and sodium hydrate in solution at 50% *w*/*w* were supplied by Panreac applique (Castellar del Vallès, Barcelona, Spain). Bis(2,4-dicumylphenyl) pentaerythritol diphosphite (Doverphos S-9228) (Dover chemical, Davis Road NW, Dover, OH, USA) and >99% methyl trichloride (chloroform) were purchased from Merck (Darmstadt, Germany). They were all reagents of analytical grade. Water was purified using a Milli-Q system (Millipore, Bedford, MA, USA). Before analysis, every solution was prepared for HPLC and purified employing a 0.45 µm polyamide filter.

### 3.2. Collection of Wastewater Samples

The PP production process is a multifaceted system that spans from the procurement of raw materials to the attainment of the final product. It commences with the reception of diverse elements, including propylene, catalysts, hydrogen, co-catalysts, selectivity control agents, and nitrogen. These elements form the basis for the initial stage of the process, where the polymerization and synthesis of PP occur within a dedicated reactor. Upon the materials reacting within the reactor, the unutilized propylene undergoes recovery in the polymerization stage. The subsequent phase involves delivering the newly formed polymer to a purging column. This column purifies the polymer by removing volatile organic compounds—a process facilitated by applying nitrogen. 

Throughout the process, an array of chemicals is added to enhance and complement the properties of PP, thereby optimizing its quality and applicability. This addition of additives is considered crucial for tailoring the product’s final characteristics to suit its intended uses. Once these stages are completed, the PP, with its improved properties, undergoes extrusion and granulation. During extrusion, aqueous condensates are generated, which, upon accumulation, transform into industrial wastewater. These wastewater streams carry a significant load of organic material and are critical to consider. For this specific investigation, meticulous sampling was conducted on these industrial wastewater streams. Sampling was performed every 12 min for 4 h, obtaining about five samples per hour of 200 mL each for 20 samples per day (see Figure 1a). 

These samples were carefully maintained in amber glass bottles at a controlled temperature of 4 °C to preserve their integrity and composition. It is important to note that due to the large-scale production and efficiency of the process, a substantial volume of PP, approximately 30 tons, can be processed within a relatively short timeframe—around 50 min. At this point, it should be clarified that the volume of samples collected is necessary to generate statistically reliable data at the laboratory scale, given the efficiency and large-scale nature of the process. However, this amount of samples is not a representative set for the industrial scale.

### 3.3. Description of the Analyte of Interest Extraction Method

Before retrieval, the material being examined undergoes passage through a polytetrafluoroethylene (PTFE) filter with a porosity of 0.22 µm to reduce the difficulty of the preparation of the sample in the following steps and to inhibit or reduce the proliferation of any microorganisms that may be present in the sample. This pretreatment procedure is also employed to remove the inorganic load from the sample, thus facilitating solid-phase extraction (SPE) and extending the lifespan of SPE cartridges. In the SPE process (see Figure 1b), the stationary phase ligands of the X-33 cartridge (500 mg, 6 mL) are initially conditioned and activated by adding 5 mL of methanol. The stationary phase of the cartridge is then equilibrated by adding 5 mL of purified H_2_O. Consequently, to retain the target analyte in the cartridge’s solid phase, 10 mL of sample is added at a rate of 1 mL min^−1^. After that, MeOH:H_2_O 80:20 is used to wash away all of the stationary phase’s contaminants. Finally, the analyte of interest is separated from the stationary phase of the cartridge with 10 mL of ACN. The cartridges were regenerated by a cleaning process involving the addition of 5 mL of purified water to elute the acetonitrile fully. Subsequently, the activation, equilibration, and other relevant steps were repeated using the solvents above. At this point, SPE has already been performed. However, an additional step is required for this investigation; this step consists of stripping the analyte of the solvent to obtain it in a solid state, which is performed by flowing pressurized N_2_ gas with a magnitude of 5 psi. To perform HPLC analysis, the extracted solid is dissolved in 1 mL of ACN [63]. The pre-concentration process was repeated until 500 mL of concentrate was generated. The recovered analyte of interest is stored inside the cabin with controlled humidity and heat for further characterization by FTIR, UV–Vis, DSC, MS, and TGA. After characterization, this BDPD is added at 0.1% to the virgin PP resin.

### 3.4. Doverphos Separation, Quantification, and Fragmentation System by HPLC-DAD/MS/MS/MS

This investigation utilizes an analytical setup comprising an Agilent (Santa Clara, CA, USA) 1200 High-Performance Liquid Chromatography (HPLC) system combined alongside a Micromass Quattro II triple quadrupole mass spectrometer. Spectral data are acquired through both MS (Mass Spectrometry) and MS/MS (Tandem Mass Spectrometry) modes. The experimental setup encompasses a Lichrosorb RP-18 column with dimensions of 4.6 × 200 mm as well as a 5-micron particle size, alongside auxiliary components such as a degasser (G1322A), a quaternary pump (G1311A), an automated sampling system (G1313A), and a column carrier (G1316A). Chromatographic parameters are established through automated injection of the extracted solution. The separation methodology involves the utilization of diverse proportions of acetonitrile (ACN) and water (H_2_O) in varying compositions: 100 percent ACN plus 0 percent H_2_O (8 min, flow rate 3.5 mL min^−1^), 84 percent ACN plus 16 percent H_2_O (1 min, flow rate 15 mL min^−1^), 92 percent ACN and 8 percent H_2_O (2 min, flow rate 2 mL min^−1^), and 96 percent ACN and 4 percent H_2_O (3.5 min, flow rate 3.5 mL min^−1^). For additive identification, MS fragment ion mass data (*m*/*z*) 191, 228, 353, 417, 435, and 549 are used [26,53,54,55,56,57,58,59,60,61,72,73,74,75].

### 3.5. Fourier Transform Infrared (FTIR)

The Nicolet (Green Bay, WI, USA) 6700 Fourier Transform Infrared (FTIR) spectrometer utilization, characterized by a sensitivity of 2 cm^−1^ and a spectral range spanning from 600 to 4000 cm^−1^, is fundamental in this analysis. Employing an attenuated total reflectance (ATR) configuration operating in absorbance mode, this system delivers spectra derived from an average of 20 scans, ensuring statistical robustness and reliability in the data acquisition process. The FTIR investigation encompasses a diverse array of samples, encompassing the examination of the recovered BDPD, the pure additive as the standardized reference, and the combinations of PP with these additives. Specifically, the focus lies on two scenarios: PP blended with the recovered additive and PP amalgamated with the pure additive. This meticulous and comprehensive approach enables a thorough comparative evaluation of spectral characteristics within these compounds, facilitating the discernment and distinction of their chemical attributes and intermolecular interactions.

### 3.6. Ultraviolet–Visible Spectroscopy (UV–Vis)

The analytical approach in this investigation involves the utilization of a single-beam UV–Vis Evolution 60S Thermo Scientific (Waltham, MA, USA) spectrometer, configured to scan wavelengths varying from 190 to 800 nm with a 2 nm resolution. Chloroform serves as the solvent for the sample preparations while maintaining a controlled temperature of 35 °C and applying ultrasound at a frequency of 60 Hz to enhance the solubility of the analyte within the solutions. The UV–Vis spectroscopic analysis is conducted on two primary components: the recovered BDPD and the pure additive. By subjecting these substances to UV–Vis analysis under controlled conditions, the aim is to elucidate and compare their absorbance characteristics across the specified spectral range. This approach aids in understanding the electronic transitions and absorption properties inherent to these compounds, providing valuable insights into their chemical structure and behavior in solution.

### 3.7. Thermogravimetric Analysis (TGA)

Thermogravimetric analysis (TGA) is carried out by the use of a Perkin Elmer (Shelton, CT, USA) TGA7 thermobalance, which covers a temperature range from 51 to 838 °C with a controlled heating rate of 20 °C/min. The carrier gas employed during the analysis is nitrogen (N_2_) with a precisely regulated gas transfer rate of 0.83 ± 0.005 mL/s. The initial deformation temperature during the TGA process was determined. This fundamental parameter provides information on the stability to withstand the heat and degrading characteristics of the analyzed substances. The analysis is performed on two main components (blends): recovered BDPD plus PP and pure additive plus PP. By subjecting these materials to TGA, the objective is to evaluate their thermal behavior and decomposition patterns, and to discern any variation in their thermal stability when combined with PP.

### 3.8. Oxidation Induction Time (OIT)

The determination of the oxidation induction time (OIT) was performed using the DSC Q2000 V24.11 Build 124 apparatus, which was specifically designed on account of calorimetric studies, and is used to determine the oxidation induction time (OIT). For the evaluations, which were carried out in nitrogen and oxygen gaseous atmospheres, 6.6 mg of sample was employed. The use of nitrogen provided a controlled and inert environment, crucial for examining its influence on the decomposition behavior of PP. The experimental procedure commenced with an isothermal phase set at 60 °C for 5 min, employing a nitrogen atmosphere at a flow rate of 50 mL min^−1^. This phase aimed to establish a baseline understanding of PP decomposition within an inert atmosphere. Subsequently, the environmental conditions were modified, subjecting the sample to an escalating temperature from 60 °C to 200 °C over 20 min and undergoing an oxidizing atmosphere of 50 mL min^−1^ airflow. This oxidation phase was sustained for 30 min at 200 °C, enabling the observation of the oxidative effects on the sample. It showed a noticeable change in the exothermic heat slope during the oxidation flow, which suggests that oxidation had started in the sample. The determination of the OIT involved identifying the moment when this slope alteration occurred. This transformation provided critical information regarding the duration required for PP to undergo oxidation when combined with additives. This analysis aids in evaluating the effectiveness of the additives in enhancing the oxidative stability of PP.

### 3.9. Mixing of the Recovered Bis(2,4-Dicumylphenyl) Pentaerythritol Diphosphite and PP Resin

Samples comprising virgin PP, recovered Bis(2,4-dicumylphenyl) pentaerythritol diphosphite, and the pure additive are individually processed in a Prodex Henschel 115JSS standard mixer. At room temperature and 800 rpm, the mixing procedure takes seven minutes. These mixes’ concentrations are kept below the theoretical threshold of 0.1%. Subsequently, these blended compositions undergo extrusion within a Welex-200 24.1 extruder (Graham Engineering Company, 1203 Eden Road, York, PA 17402 USA). The temperatures of operation for the extrusion process range from 190 to 220 °C. This phase aims to assess the behavior and characteristics of these blends under varied extrusion conditions. Following extrusion, the prepared blends are molded into sheets using a compression molding technique employing the CARVER (Coeymans, NY, USA) 3895 hot press. This molding process facilitates the transformation of the blended compositions into uniform sheets, allowing for subsequent analysis and characterization of their properties and performance.

## 4. Conclusions

The methodology was validated to quantify Doverphos in wastewater. The method developed by HPLC presented an RSD, Er, linearity, and % recovery that were less than 2.6, 2.5, more significant than 0.9995, and greater than 96%, respectively, for inter-day and intra-day tests of the chromatographic method and SPE. The chromatographic methodology showed LOQ from 5.4 to 16 mg L^−1^ and LOD between 1.3 and 7.7 mg L^−1^. Doverphos was found in the wastewater at concentrations between 1179.0 and 4709.6 mg L^−1^. Doverphos is extracted from industrial wastewater with a purity of 95% and was characterized by FTIR, UV–Vis, DSC, TGA, and MS, which allowed elucidation of the structure of bis(2,4-dicumylphenyl) pentaerythritol diphosphite. The recovered OPE was mixed with PP resins, which allowed it to improve its thermal properties and minimize its thermo-oxidative degradation.

In conclusion, this study emphasizes the substantial environmental impact achievable through the recovery of BDPD from industrial wastewater in polymer production. The concentrations of organophosphorus flame retardants (OPEs) in wastewater highlight the urgent need for global attention to mitigate potential environmental and health risks. The search for environmentally acceptable alternatives in petrochemical processes is being conducted to reduce the harmful effects of the worrisome amounts of OPEs in wastewater on human health and ecosystems. This is a cause for international concern. This study drives continuous efforts in the polymer sector to promote sustainable growth and ethical business practices.

## Figures and Tables

**Table 1:** Range of values recorded for physicochemical parameters of the collected samples.

Parameter	Interval	Unit
Temperature	35.1–38.9	°C
pH	6.77–8.31	
Chemical oxygen demand (COD)	23.4–35.8	mg/L O_2_
Biochemical oxygen demand (BOD5)	5.2–8.3	mg/L O_2_
Total Suspended Solids (TSS)	12.3–35.9	mg/L
Sedimentable Solids	1.1–1.6	mg/L
Aluminium (Al)	0.0001–0.0003	mg/L
Cadmium (Cd)	Not detected	mg/L
Zinc (Zn)	Not detected	mg/L
Copper (Cu)	0.1–0.3	µg/L
Chrome (Cr)	Not detected	mg/L
Iron (Fe)	0.015–0.024	mg/L
Mercury (Hg)	Not detected	mg/L
Nickel (Ni)	Not detected	mg/L
Silver (Ag)	0.001–0.004	µg/L
Lead (Pb)	Not detected	mg/L
Chloride (Cl^−^)	0.015–0.023	mg/L
Sulfates (SO_4_^2−^)	0.0014–0.0037	mg/L
Phosphates (PO_4_^3−^)	0.0027–0.0045	mg/L
Sulfides (S^2−^)	0.0011–0.0021	mg/L
Nitrites (NO_2_^−^)	0.046–0.0089	mg/L
Nitrates (NO_3_^−^)	0.068–0.0335	mg/L

**Table 2:** Data obtained for the calibration curve of Bis(2,4-dicumylphenyl) pentaerythritol diphosphite.

Inter-Day				Intra-Day			
Abstract (mg/L)	Experimental ± s (mg/L)	RSD (%)	Er (%)	Abstract (mg/L)	Experimental ± s (mg/L)	RSD (%)	Er (%)
1000	985.0 ± 21.5	2.2	1.5	1000	966.0 ± 20.4	2.1	3.4
1500	1492.2 ± 14.6	1.0	0.5	1500	1487.8 ± 15.7	1.1	0.8
2000	1954.6 ± 51.7	2.6	2.3	2000	1973.2 ± 12.2	0.6	1.3
3000	2926.2 ± 53.7	1.8	2.5	3000	2947.6 ± 49.1	1.7	1.7
5000	4981.0 ± 9.3	0.2	0.4	5000	4965.2 ± 20.0	0.4	0.7

**Table 3:** Extraction data of Bis(2,4-dicumylphenyl) pentaerythritol diphosphite by SPE.

Inter-Day					Intra-Day				
Abstract (mg/L)	Experimental ± s (mg/L)	RSD (%)	Er (%)	Recovery (%)	Abstract (mg/L)	Experimental ± s (mg/L)	RSD (%)	Er (%)	Recovery (%)
1000	958.6 ± 19.4	2.0	4.1	96	1000	966.0 ± 20.4	2.1	3.4	97
1500	1462.8 ± 33.3	2.3	2.5	98	1500	1487.8 ± 15.7	1.1	0.8	96
2000	1913.2 ± 44.6	2.3	4.3	96	2000	1973.2 ± 12.2	0.6	1.3	97
3000	2934.2 ± 69.3	2.4	2.2	98	3000	2947.6 ± 49.1	1.7	1.7	97
5000	4897.0 ± 78.7	1.6	2.1	98	5000	4965.2 ± 20.0	0.4	0.7	97

**Table 4:** Estimating the R^2^ and r for RPED and RPOD from the calibration curve and SPE.

Values and Parameters for R^2^ and r for Doverphos S-9228				
Equation	$y=mx+b\;\;\left(1\right)$			
Plot	Calibration Curve Repeatability	Calibration Curve Reproducibility	SPE Repeatability	SPE Reproducibility
Intercept	−13.34247 ± 19.00663	−12.54247 ± 10.63192	−16.39178 ± 13.19924	−8.61096 ± 8.4511
Slope	0.99352 ± 0.00725	0.9932 ± 0.00405	0.98113 ± 0.00503	0.9738 ± 0.00322
Residual Sum of Squares	3196.53304	1000.21304	1541.58312	631.96822
Pearson’s r	0.99989	0.99997	0.99995	0.99998
R-Square (COD)	0.99979	0.99993	0.99989	0.99996
Adj. R-Square	0.99973	0.99992	0.99987	0.99995

**Table 5:** Summary of the risks and benefits of BDPD.

Potential Risks and Benefits of BDPD	Damage Classifications	Source
Affectation of the endocrine system, with hormonal changes. Dysfunction in the body’s metabolic system, modifying molecular mechanisms. Damage and dysfunction of the cardiovascular system and liver tissue. Symptoms such as headaches, anxiety, confusion, and alterations in consciousness. Possible adverse effects on neurological development in children. Presence of OPE in the placenta of pregnant women, with potential adverse effects on fetal development.	Human health	[34,35]
Could have detrimental long-term consequences on aquatic life.	Environments	[35,45,46,47]
Reduction in production costs (millions of dollars).	Resources	Not Applicable

## Data Availability

The data are partially available due to confidentiality at the chemical plant where the evaluations were carried out.
